# LDL-receptor gene polymorphism as a predictor of coronary artery disease: an Egyptian pilot study: relation to lipid profile and angiographic findings

**DOI:** 10.1186/s43044-023-00430-w

**Published:** 2024-01-02

**Authors:** Kefaya El-Sayed, Amany R. Youssef, Nehal M. Abdel Hay, Adel M. Osman

**Affiliations:** 1 Department of Clinical Pathology, Faculty of Medicine, Mansoura University, Mansoura, 35516 Egypt; 2 Faculty of Medicine, Mansoura University, Mansoura, Egypt; 3 Cardiology Department, Faculty of Medicine, Mansoura University, Mansoura, Egypt

**Keywords:** CAD, rs1122608, Genetic polymorphism, LDL receptor

## Abstract

**Background:**

Coronary artery disease (CAD) is the main cause of death in Egypt. Many LDL-R gene locus single nucleotide polymorphisms (SNP) are found to be associated with the risk of CAD. This research aimed to assess the allelic and genotypic frequencies of rs1122608 SNP and their association with the extent of vessel affection and lipid profile in a population of Egyptians.100 CAD patients and 100 healthy controls of Egyptians were included. PCR–RFLP was used to genotype rs1122608 SNPs.

**Results:**

Significantly higher proportion of ‘T’ allele among patient (risk allele). This association is of low strength (ϕ lies between 0.1 and 0.3). A participant with ‘T’ allele has 1.95 times higher odds to exhibit CAD versus a participant with ‘G’ allele. Significantly higher proportion of ‘T/T’ genotype among cases versus control (risk genotype). This association is of low strength (Cramer’s V lies between 0.1 and 0.3). A participant with ‘T/T’ genotype has 4.5 times higher odds to exhibit CAD versus a participant with ‘G/G’. Gensini score showed no significant association with rs1122608 genotypes (*p* = 0.863).

**Conclusions:**

The mutant GT and TT genotypes and minor T allele of rs1122608 were positively correlated with CAD and considered as independent risk factors for CAD.

## Background

One of the most reasons of morbidity and death for both men and women across practically all racial and ethnic groupings is coronary artery disease (CAD) [1]. Rapid urbanization, sedentary lifestyles, changing eating habits, and increased levels of stress in emerging nations are all contributing to an alarming increase in the prevalence of CAD [2]. CVD accounted for 46.2% of deaths overall in 2017 [3]. CAD prevalence among Egyptians is 8.3% [4]. Egyptian fatalities from CAD accounted for 29.38% of all deaths in 2018, according to WHO statistics [5]. However, little is known about the pathophysiology of CHD. However, coronary heart disease (CHD) is believed to be a polygenic illness brought on by the combination of hereditary and environmental factors. Elevated plasma levels of low-density lipoprotein cholesterol (LDL-C) are a major contributor to the occurrence of coronary events, according to epidemiological and clinical studies [6]. The lipid profile may be harmed by LDL-R malfunction and LDL-R expression and dysregulation [7]. Single nucleotide polymorphisms (SNPs) in the LDL-R gene locus have been linked to CAD risk in genome-wide association investigations [8, 9]. The LDL-R gene upstream locus, rs1122608-G/T, has been shown to be related with CAD risk [10]. The SWI/SNF-related matrix-associated Actin-dependent regulator of chromatin, subfamily A, member 4 (SMARCA4), also known as BRG1, has the single nucleotide polymorphism (SNP) rs1122608 in intron 30, which is close to the LDL-R gene. The protein SMARCA4 is a crucial catalytic constituent of the SWI/SNF (switching defective/sucrose non-fermenting) complexes, which are in the 19p13.2 region of the chromosome [11]. Utilizing the chemical energy of adenosine triphosphate hydrolysis, SWI/SNF alters the structure of chromatin and controls the transcription of several genes [12]. The rs1122608 most likely modifies SMARCA4 expression [13]. Despite evidence linking the SMARCA4 rs1122608 SNP to CAD risk in the Caucasian population, there is conflicting evidence in the Asian population [14]. Homozygote genotypes for the SMARCA4 rs1122608 SNP had a significant protective impact against CAD, according to one study of Iranian populations [1]. However, it is uncertain if SMARCA4 rs1122608 SNP plays a protective or risky function in the development of CAD. Furthermore, no prior reports have been made about the connection between the SMARCA4 rs1122608 SNP and CAD among Egyptians. This research aimed to assess the allelic and genotypic frequencies of rs1122608 SNP and their association with the extent of vessel affection and lipid profile in a population of Egyptians.

## Methods

### Setting

This study was done in the Specialized Medical Hospital (Cardiology Department), Mansoura University, Egypt, between November 2019 and December 2021.

### Sample size

Sample size was calculated by using the online Genetic Association Study (GAS) Power Calculator, 2017 Jennifer Li Johnson, University of Michigan School of Public Health. Based on a previous study by Myocardial Infarction Genetics Consortium (2009) [15], a large-scale study requires 1000 cases and 1000 control subjects to achieve 97.3% expected power for a one-stage study at 0.001 significance (α) level to detect a disease allele frequency of 0.75, and genotype relative risk of 1.5 for CAD assuming 0.1 disease prevalence. Accordingly, this pilot study, using a rule of thumb, was conducted on 10% of the required sample size for a large-scale study, i.e., 100 cases versus 100 control subjects.

### Inclusion criteria

#### Case

Patients who had atherosclerotic CAD were evaluated using standardized coronary angiography in accordance with Seldinger's method [16]. According to the results of the coronary angiography, 100 CAD patients with at least one vessel having > 50% stenosis were selected. All patients signed an informed written consent.

#### Control

One-hundred age- and sex-matched healthy controls, all signed an informed written consent.

#### Exclusion criteria

Aortic dissection, severe heart failure, cardiogenic shock, malignant arrhythmia, malignancies, autoimmune illnesses, mental illnesses, patients with diabetes, hepatic, renal, or lung malfunction, and CAD patients using hormonal contraceptives were all disqualified from the study. Patients with incomplete clinical data were also excluded.

### Methodology

All participants had a thorough medical assessment in addition to having their family history of myocardial infarction, diabetes, and hypertension checked.

### Assessment of coronary arteries

The severity of coronary affection of the patients were assessed according to the Gensini score standard [17]. The examination of coronary angiograms was carried out without knowledge of the genetic status.

### Blood specimen collection and laboratory testing

The laboratory tests were performed at the clinical chemistry and molecular labs of the Specialized Internal Medicine Hospital and Oncology Center at Mansoura University in Egypt. After an overnight fast, five milliliters of blood were drawn from each participant's peripheral vein by aseptic vein puncture. Three milliliters of the blood were collected as whole blood, and the remaining milliliters were separated into serum for the biochemical studies (serum total cholesterol (TC) and triglycerides (TG) levels were determined utilizing an enzymatic method, and high-density lipoprotein (HDL-C) was determined utilizing a homogenous enzymatic direct assay using the autoanalyzer Cobas C311 (Roche diagnostics international, USA). The Friedewald formula was used to determine LDL-C as TG levels were not exceeded 400mg/dl (4.5mmol/L). LDL-C (mmol/L) = (TC) − (HDL-C) − (TG/2.2) [18]. DNA was taken from two milliliters of blood and kept at − 20 °C until it was time for amplification and genotyping by PCR–RFLP [19], Sense primer 5′-GAACGCCCCTCAAGCTGCCCTCC-3 and antisense primer 5′-AGCCACCGTGCCCAGCC-TCCAA-3 were utilized for the amplification of the area harboring the polymorphism of SMARCA4 rs1122608. The reaction volume was 25 μL and the components were as follows: 11.5 μL of ddH2O (DNase/RNase-free), 12.5 μL of Crystal Taq Master Mix (Jena Bioscience, Germany), 0.1 μL of each primer, and 1.0 μL of gDNA. Denaturation at 95 °C for 2 min, followed by 35 cycles of 95 °C for 30 s, 68.5 °C for 30 s, and 72 °C for 30 s, were the thermocycling conditions for the PCR. After these 35 cycles, there was a 7-min extension step at 72 °C. The PCR products were then digested with BsrI at 65 °C for 3 h (Thermo scientific, Lithuania). For each reaction, the following ingredients were introduced. 8.5 μl of nitrous-free water, 1 μl of buffer, 0.5 μl of BsrI, and 5 μl of PCR products. After the amplified gDNA underwent restriction digestion, the fragments were separated by electrophoresis using a 50 bp DNA ladder on 2% agarose gels containing ethidium bromide and observed under ultraviolet light to determine the genotypes. An expert reader who was blinded to the blood cholesterol levels and epidemiological information scored the genotypes. The detected genotypes were given names based on whether restriction sites were present (G allele) or absent (T allele), with the GG genotype being known as the wild-type homozygote. The lack of the restriction site was the defining characteristic of this wild-type GG genotype (with 280 and 139 bp bands). Both the lack and presence of the restriction site were present in the heterozygotic GT genotype (280, 139, 211, 74, and 211 bp bands). Only the presence of the mutant site (139, 211, and 74 bp bands) was present in the homozygotic TT genotype.

## Results

### Statistical analysis

Data were entered and analyzed by using SNP Stats software: https://www.snpstats.net/start.htm.

This involves allele frequencies, Genotype frequencies, Hardy–Weinberg equilibrium (HWE), and analysis of association with CAD. Data were also entered and analyzed using IBM-SPSS software (IBM Corp. Released 2017. IBM SPSS Statistics for Windows, Version 25.0. Armonk, NY: IBM Corp.). Qualitative data were expressed as N (%) and chi-Square test was used to compare categorical data. Quantitative data were initially tested for normality using Shapiro–Wilk’s test with data being normally distributed if *p* > 0.050. Presence of significant outliers was tested for by inspecting boxplots. Quantitative data were expressed as mean ± standard deviation. Quantitative data between two groups were compared by Independent-Samples t-test. For any of the used tests, results were considered as statistically significant if *p* value ≤ 0.050.

## Results

This study involved 200 participants (100 CAD cases and 100 age- and sex matched controls).

Table 1 shows that CAD and control groups did not differ significantly as regards age and sex, residence, and HDL-C level and differ significantly as regards all other parameters.

**Table 1:** Characteristics of CAD patients versus control group

Parameter		Control group (n = 100)	CAD group (n = 100)	*p*
Age (years)		56.3 ± 8.3	58 ± 7.9	0.150
Gender	Male	69 (69%)	79 (79%)	0.107
	Female	31 (31%)	21 (21%)	
Residence	Rural	47 (47%)	35 (35%)	0.084
	Urban	53 (53%)	65 (65%)	
Smoking		14 (14%)	57 (57%)	< 0.001
Hypertension		4 (4%)	96 (96%)	< 0.001
BMI (kg/m^2^)		26.1 ± 4.6	28.7 ± 3.1	< 0.001
Total cholesterol (mmol/L)		4.6 ± 0.66	6.18 ± 0.75	< 0.001
TG (mmol/L)		1.14 ± 0.37	1.62 ± 0.29	< 0.001
HDL-C (mmol/L)		1.34 ± 0.27	1.27 ± 0.31	0.112
LDL-C (mmol/L)		2.79 ± 0.63	4.15 ± 0.74	< 0.001

Categorical data are N (%) and are compared by chi-square test while the numerical data are mean ± SD and are compared by independent-samples t-test

### Exact test for Hardy–Weinberg equilibrium (HWE) [n = 200]

All subjects, controls, and cases were in HWE, *p* values = 0.56, 0.64, and 1.00, respectively.

Conducting multivariable analysis on significant confounders revealed that higher frequency of hypertension, GT, TT, GT + TT genotype, T allele, unhealthy dietary pattern and higher levels of BMI, TC were considered independent predictors of CAD susceptibility. While, healthy dietary pattern is considered protective against CAD (*p* < 0.021, OR = 0.933, 95% CI 0.880–0.990).

## Discussion

For many years, research has focused heavily on the crucial role that lipid regulation genes play a role in the etiology of CAD. Previous research works studied the relation of SMARCA4 rs1122608 SNP and risk of CHD in Caucasians and Asians. In the present study we investigated SMARCA4 rs1122608 SNP in CAD in a group of Egyptian patients Uncertainty exists about the processes behind the SMARCA4 rs1122608 SNP's contribution to the increased risk of CAD. By altering the structure of chromatin with the help of the chemical energy of adenosine triphosphate hydrolysis, SMARCA4 controls the transcription of many genes. Earlier Expression Quantitative Trait Locus (eQTL) studies suggested that rs1122608 is probably modulating SMARCA4 expression [13]. According to our findings, CAD was substantially related with the mutant GT and TT genotypes as well as the minor T allele of the SMARCA4 rs1122608 SNP (Table 2).

**Table 2:** Allele frequencies in cases versus the control

Allele	N (%)			Chi-square test			Binary logistic regression		
	Control	Case (CAD)	Total	χ^2^	Phi (ϕ)	*p* value	COR	95% CI	*p* value
G	60 (30%)	36 (18%)	96 (24%)	7.895	0.140	**0.005**	r(1) 1.95	r(1) 1.2–3.1	**0.005**
T	140 (70%)	164 (82%)	304 (76%)						

Bold value indicate significance *p* ≤ 0.050

*CAD* coronary artery disease, *COR* crude odds ratio, *CI* confidence interval

r(1) = reference category

This table shows a statistically significantly higher proportion of ‘T’ allele among cases versus control (risk allele). This association is of low strength (ϕ lies between 0.1 and 0.3). A participant with ‘T’ allele has 1.95 times higher odds to exhibit CAD versus a participant with ‘G’ allele

Coronary angiography is the gold standard to evaluate the severity of CAD. We evaluated a possible contribution of rs1122608 SNP to CAD by accredited coronary scoring system. However, there was no interaction between these rs1122608 variants and extent of vascular affection and Gensini score of CAD patients (Table 5). Chen et al. [19], revealed that the minor T allele of rs1122608 SNP and the mutant GT and TT genotypes were strongly linked with the risk of AMI in Chinese individuals. rs1122608 SNP was shown to be a risk variation for CHD in Europeans [20] and Caucasians but not in Asians in a meta-analysis research [21]. Jamaldini et al. [1], revealed that rs1122608 (GT or TT) was at greater risk of three vessel compared to single vessel affection, with less frequency of TT genotype in CHD group in Iranians. Guo et al. [22], revealed no association between rs1122608 with CHD in Chinese patients. Different allele frequencies and other variables, such as lifestyle differences, may be to blame for this racial variation occurrence; moreover, SMARCA4 may function differently in varying disease mechanism [23]. SMARCA4 rs1122608 SNP involvement in elevation of CHD risk is in need to be clarified. The large, ATP-dependent SWI/SNF chromatin remodeling complex's catalytic component is encoded by SMARCA4. This complex affects transcriptional control in an ATP-dependent way by breaking down histone-DNA interactions, which is necessary for the transcriptional activation of genes typically suppressed by chromatin. Numerous studies have shown that SMARCA4 may influence the development of AMI by controlling vascular smooth muscle [24, 25].In the current research, there was no substantial association between the present dyslipidemia in CAD patients and SMARCA4 rs1122608 SNP genotypes (Table 6). Numerous studies have shown that SMARCA4rs1122608 is linked to CAD irrespective of lipid profiles [26], since it sits next to LDL-R but not inside it [20]. Martinelli et al. [20] reported that LDL-R may affect both plasma lipids and the concentration of coagulation factors thus, modulating the risk of CAD. They reported that the function of LDL-R is not limited to lipoprotein metabolism, so it acts as a balance for hemostasis and clotting factors. CHD involves a variety of environmental and behavioral risk factors [27]. Although many environmental variables have been shown to affect biological processes, surprisingly little is understood about how genes and the environment interact. Studies on the interactions between genes and the environment reveal that a person's diet may affect their genetic susceptibility to cardiovascular risk. The processes behind the interaction between a modifiable factor and the genetic background are still unknown; gene-diet interaction studies aim to shed light on this interaction. The goal of epigenetic research is to clarify the biological mechanisms impacted by environmental factors, such as food exposure [28]. The combined genetic and environmental effects of rs1122608 have been attributed to telomere shortening [29]. In agreement with our study, Abudureyimu et al. [30] found no association between the SNP rs 1122608 and the angiographic pattern of CAD patients. They reported that rs1122608 variants were not related to CAD in multivariate analysis. Allele frequency and other variables, such as lifestyle differences, may be to blame for this racial difference phenomenon. Additionally, SMARCA4 may operate differently in various disease mechanisms [24] (Tables 3, 4, 5, 6, 7).

**Table 3:** Genotype frequencies in case versus control

Genotype	N (%)			Chi-square test			Binary logistic regression		
	Control	Case (CAD)	Total	χ^2^	Cramer’s V	*p* value	COR	95% CI	*p* value
G/G	10 (10%)	3 (3%)	13 (6.5%)	7.668	0.196	**0.022**	r(1) 2.5 4.5	r(1) 0.63–9.9 1.2–17.1	0.191 **0.029**
G/T	40 (40%)	30 (30%)	70 (35%)						
T/T	50 (50%)	67 (67%)	117 (58.5%)						

Bold value indicate significance *p* ≤ 0.050

*CAD* coronary artery disease, *COR* crude odds ratio, *CI* confidence interval

r(1) = reference category

This table shows a statistically significantly higher proportion of ‘T/T’ genotype among cases versus control (risk genotype). This association is of low strength (Cramer’s V lies between 0.1 and 0.3). A participant with ‘T/T’ genotype has 4.5 times higher odds to exhibit ACS versus a participant with ‘G/G’ genotype

**Table 4:** SNP association with CAD (n = 200, adjusted by age and sex)

Model	Genotype	Control	Case (CAD)	AOR (95% CI)	*P* value	AIC	BIC
Co-dominant	T/T	50 (50%)	67 (67%)	r(1)	0.0077	243.8	260.3
	T/G	40 (40%)	30 (30%)	2.61 (0.6–11.3)			
	G/G	10 (10%)	3 (3%)	**5.6 (1.3–23.6)**			
Dominant	T/T	50 (50%)	67 (67%)	r(1)	0.0049	243.6	256.8
	T/G-G/G	50 (50%)	33 (33%)	**4.2 (1.02–16.9)**			
Recessive	T/T-T/G	90 (90%)	97 (97%)	r(1)	0.033	247	260.2
	G/G	10 (10%)	3 (3%)	**2.45 (1.30–4.6)**			
Over-dominant	T/T-G/G	60 (60%)	70 (70%)	r(1)	0.07	248.2	261.4
	T/G	40 (40%)	30 (30%)	0.55 (0.29–1.06)			
Log-additive	–	–	–	**2.24 (1.32–3.80)**	0.0019	241.8	255

Bold value indicate significance *p* ≤ 0.050

*CAD* coronary artery disease, *AOR* adjusted odds ratio, *CI* confidence interval, *AIC* Akaiki information criterion, *BIC* Bayesian information criterion

r(1) = reference category

This table shows that log-additive model is the best inheritance model (with the lowest p-value, AICC, and BIC). Participants with ‘T’ allele have 2.2 times higher odds adjusted for age and sex to exhibit CAD compared to participant with ‘G’ allele

**Table 5:** Association of rs1122608 genotype and severity of CAD patients

Cases		GG	GT	TT	*P*
		n = 3	n = 30	n = 67	
EF (%)		57 ± 9.5	58 ± 12.5	55.4 ± 10.8	0.816
ECG	Average	1 (33.3%)	18 (60%)	37 (55.2%)	0.682
	Abnormal	2 (66.7%)	12 (40%)	30 (44.8%)	
Uni vessel N = 57		2 (3.5%)	18(31.6%)	37 (64.9%)	0.378
Two vessels N = 31		1 (3.2%)	11 (35.5%)	19 (61.3%)	
More than two vessels N = 12		0 (0.0%)	1 (8.3%)	11 (91.7%)	
Gensini score		26.7 ± 19.7	32 ± 30.1	34.4 ± 29.5	0.863

This table reveals no significant differences found regarding ejection fraction (EF), electrocardiograph (ECG) among rs1122608 genotypes in CAD group (*p* = 0.816, 0.682 respectively), no significant association is found between rs1122608 genotypes and alleles with extent of vessel affection (*p* = 0.378), in spite of increased frequency of TT genotype in CAD patients with multi-vessel affection more than those with one vessel and two vessel affected (91.7%, 61.3%, 64.9% respectively). As regard Gensini score, it shows no significant association with rs1122608 genotypes (*p* = 0.863)

**Table 6:** Association of rs1122608 genotypes and serum lipid levels in CAD patients

Cases	GG	GT	TT	*P*
	n = 3	n = 30	n = 67	
Total Cholesterol (mmol/L)	6.42 ± 0.99	6.22 ± 0.70	6.16 ± 0.77	0.796
TG (mmol/L)	1.84 ± 0.12	1.64 ± 0.31	1.60 ± 0.29	0.374
HDL-C (mmol/L)	1.13 ± 0.36	1.31 ± 0.32	1.26 ± 0.31	0.605
LDL-C (mmol/L)	4.43 ± 0.74	4.14 ± 0.75	4.15 ± 0.75	0.809

As shown in this table, no significant differences are found regarding serum lipid levels (TC, TG, HDL-C, LDL-C) among genotypes in CAD group (*p* = 0.796, 0.374, 0.605, and 0.809 respectively).

**Table 7:** Regression analysis for prediction of CAD susceptibility

	Univariable				Multivariable			
	*P*	OR	95% CI		*p*	OR	95% CI	
Age	0.149	1.016	0.994	1.038				
Gender	0.107	0.720	0.483	1.074				
Smoking	**< 0.001**	3.605	2.413	5.387	0.767	1.012	0.935	1.095
BMI	**< 0.001**	1.116	1.064	1.170	**0.021**	1.008	1.001	1.014
HTN	**< 0.001**	1.885	1.486	2.390	**< 0.001**	1.874	1.722	2.040
Total Cholesterol	**< 0.001**	1.056	1.041	1.071	**0.002**	1.003	1.001	1.006
TG	**< 0.001**	1.027	1.020	1.034	0.649	1.001	0.999	1.002
HDL-C	0.111	0.988	0.973	1.003				
LDL-C	**< 0.001**	1.051	1.038	1.064	0.192	0.999	0.996	1.001
GT	**< 0.001**	4.071	2.187	7.579	**< 0.001**	1.194	1.108	1.288
TT	**< 0.001**	15.043	7.838	28.873	**0.025**	1.076	1.009	1.148
GT + TT	**< 0.001**	7.360	4.099	13.215	**0.035**	1.994	1.777	1.541
T	**< 0.001**	4.241	3.224	5.578	**< 0.001**	2.364	1.988	3.595

Bold value indicate significance *p* ≤ 0.050

As shown in this table**,** Logistic regression analysis is conducted for prediction of CAD susceptibility, using age, gender, residence, smoking, BMI, hypertension, TC, TG, HDL-C, LDL-C, rs1122608genotypes, alleles, dietary patterns as covariates. Higher frequency of smoking, hypertension, GT, TT, GT + TT, genotypes, T allele, unhealthy dietary pattern, higher levels of BMI, TC, TG, LDL-C,, lower frequency of healthy dietary pattern are considered as risk factors for CAD in univariable analysis.While, healthy dietary pattern is protective for CAD (*p* < 0.001, OR = 0.206, 95% CI 0.140–0.305)

## Conclusions

There were substantial connections between the SMARCA4 rs1122608 and CAD patients compared to the controls in both the genotypic and allelic frequencies. GT and TT genotypes of rs1122608 were associated with significant rs1122608 SNP variants. GT, TT, GT + TT, and T allele were associated with CAD and considered as independent risk factor for CAD.

### Study limitations

The study limitations include the small patient population and limited genetic analysis for one gene at the SMARCA4 genetic locus. Additional large population-based wide-scale genetic studies are needed to fully comprehend the genetic contribution of the SMARCA4 genetic locus as a risk factor for CAD.

## Data Availability

The datasets used and/or analyzed during the current study are available from the corresponding author on reasonable request.

## Abbreviations

AIC: Akaiki Information Criterion
ACS: Acute Coronary Syndrome
AMI: Acute Myocardial Infarction
AOR: Adjusted Odd Ratio
ATP: Adenosine Triphosphate
BIC: Bayesian Information Criterion
bP: Base pair
CAD: Coronary Artery Disease
CHD: Coronary Heart Disease
CI: Confidence Interval
COR: Crude Odd Ratio
DNA: Deoxyribonucleic Acid
ECG: Electro Cardiogram
EF: Ejection Fraction
eQTL: Earlier Expression Quantitative Trait Locus
GAS: Genetic Association Study
gDNA: Genomic Deoxyribonucleic Acid
HDL-C: High Density lipoprotein-cholesterol
HWE: Hardy–Weinberg Equilibrium
LDL-C: Low Density lipoprotein-cholesterol
LDL-R: Low Density lipoprotein-Receptor
OR: Odd Ratio
PCR: Polymerase Chain Reaction
RFLP: Restriction Fragment Length Polymorphism
SNP: Single Nucleotide Polymorphism
SWI/SNF: Switching defective/ Sucrose non-fermenting
TG: Triglyceride
WHO: World Health Organization
